# Changes in retinal layers in type 1 diabetes mellitus without retinopathy measured by spectral domain and swept source OCTs

**DOI:** 10.1038/s41598-021-89992-w

**Published:** 2021-05-17

**Authors:** Elvira Orduna-Hospital, Ana Sanchez-Cano, Lorena Perdices, Javier Acha, Elena María Lopez-Alaminos, Isabel Pinilla

**Affiliations:** 1 Aragon Institute for Health Research (IIS Aragon), 50009 Zaragoza, Spain; 2 Department of Applied Physics, University of Zaragoza, Pedro Cerbuna, 12, 50009 Zaragoza, Spain; 3 Department of Endocrinology, Miguel Servet University Hospital, 50009 Zaragoza, Spain; 4 Department of Ophthalmology, Lozano Blesa University Hospital, 50009 Zaragoza, Spain

**Keywords:** Eye diseases, Macular degeneration, Retinal diseases

## Abstract

To evaluate changes in inner retinal layer (IRL) thicknesses in patients with type 1 diabetes mellitus (DM1) with no diabetic retinopathy (DR) using two different optical coherence tomography (OCT) devices. Ninety DM1 and 60 healthy eyes were evaluated using spectral domain (SD)-OCT and swept source (SS)-OCT to measure changes in the retinal nerve fiber layer (RNFL), ganglion cell layer (GCL), inner plexiform layer (IPL) and inner nuclear layer (INL) thicknesses in all Early Treatment of Diabetic Retinopathy Study (ETDRS) macular areas. Functional tests were performed in both groups, including ETDRS with 100, 2.5 and 1.25% contrast, and color vision. The mean ages were 42.93 ± 13.62 and 41.52 ± 13.05 years in the diabetic and control groups, respectively. Visual acuity (VA) with ETDRS 1.25% was lower in the DM1 patients. Both ETDRS 2.5% and color vision were lower in the DM1 group but did not reach statistical significance. Retinal thicknesses in the central area and in the vertical outer areas were higher in the DM1 group. Differences were found in the IRL with no changes in the outer ones. Long-term DM1 patients with no DR maintained visual function, with a decrease in VA with 1.25% ETDRS contrast. Macular thickness measurements were higher using Spectralis SD-OCT than DRI Triton SS-OCT, and DM1 patients had a decrease in IRL thickness, especially in the GCL at the parafoveal level, generating thinning of the RNFL in the peripheral areas. There were no differences in outer retinal layer (ORL) thickness.

## Introduction

In type 1 diabetes mellitus (DM1) patients, the major cause of visual loss is diabetic retinopathy (DR); before microvascular changes exist, functional changes affect retinal cells. This occurs due to the retinal neurons lost, secondary to a neurodegenerative process. Functional neurodegeneration changes in diabetes have been proven, such as low color perception^1^, reduction in contrast sensitivity (CS)^2^, diminished dark adaptation^3^, abnormal electrophysiological tests^4^, oscillatory potential reduction in the electroretinogram (ERG) or abnormal multifocal pattern ERG^5^; defects in the visual field with concurrent defects in the retinal nerve fiber layer (RNFL) have also been described^6,7^. The development of optical coherence tomography (OCT) technology permits the quantification of different retinal layer thicknesses and volume changes. This technique allows the visualization of the retinal layers thanks to the refractive properties of each layer, their segmentation and quantification in the Early Treatment of Diabetic Retinopathy Study (ETDRS) macular areas of each retinal layer. By studying changes in the different retinal layers, we identified neurodegeneration.

Several studies have demonstrated a thinning of retinal macular thickness in diabetic patients prior to any sign of DR^8–10^, mainly due to a diminution of the ganglion cell complex (GCC), comprising the ganglion cell layer (GCL), inner plexiform layer (IPL) and RNFL^11^. We demonstrated, in a retrospective study with a long follow-up time with Spectralis SD-OCT, inner retinal layer (IRL) diminution in a diabetic population before retinopathy appears^12^. Sohn et al.^13^ demonstrated RNFL and GCL-IPL loss during 4 years of follow-up using Stratus OCT in DM1 patients with no or minimal diabetic lesions and its correlation with animal models and postmortem immunohistochemistry.

The purpose of our study was to assess IRL and outer retinal layer (ORL) thickness changes with two different OCT devices, spectral domain (SD)-OCT and swept source (SS)-OCT, in the macular areas of the ETDRS in DM1 patients without retinopathy compared with a control group and to correlate the thickness results with functional findings measured by visual acuity (AV), CS or color vision in the DM1 group.

## Methods

We undertook a prospective study during 2017 including 90 eyes from 90 DM1 patients with no DR and 60 eyes from 60 age-matched healthy subjects. The experimental protocol was approved by the local Ethics Committee for Clinical Research of Aragon (CEICA 18/2017), and the evaluation was conducted in accordance with the principles of the Helsinki Declaration. Detailed consent forms were obtained from each participant.

DM1 patients were controlled by the endocrinology unit. Blood samples were analysed every six months. Glycosylated haemoglobin (HbA1c), lipid values and arterial blood pressure were maintained under extreme control.

The inclusion criteria for the DM1 group were DM1 diagnosis with no retinal changes identified by biomicroscopy or structural OCT; all subjects, the DM1 and the control group, had a best corrected visual acuity (BCVA) over 20/25 on the Snellen chart, with refractive errors between + 5.00 and − 5.00 diopters, normal anterior pole examination with slit-lamp and no fundoscopy anomalies. The control group included healthy age-matched subjects to the DM1 group.

Exclusion criteria for both groups were the presence of any sign of DR, glaucoma or intraocular pressure (IOP) over 21 mmHg assessed by Goldman tonometry, optic nerve pathology, ocular inflammation or previous ocular surgery or procedure including laser therapy, ocular traumatism, anterior segment pathology or media opacification.

At each patient’s visit, a detailed familiar, systemic and ophthalmological medical history was performed.

The axial length (AL) was measured with the optical biometry *IOLMaster 500* (Carl Zeiss Meditec, Oberkochen, Germany).

**Figure 1:**
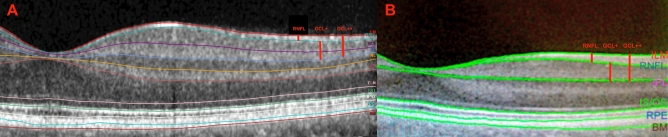
(Tomographic profile obtained by different OCT devices. (**A**) Tomographic image obtained by Spectralis SD-OCT and automatic segmentation was made by its software version 6.8.1.0. On the right margin are the abbreviations of all the layers of the automated macular segmentation provided by Spectralis SD-OCT software. The thickness of the RNFL, GCL+ and GCL++ protocols are described in the Materials and Methods section. Retinal nerve fiber layer thickness (RNFL) from the inner limiting membrane (ILM) line to the RNFL line. GCL+ thickness from the RNFL line to the inner plexiform layer (IPL) line and GCL++ thickness from the ILM line to the IPL line. GCL, ganglion cell layer; INL, inner nuclear layer; OPL, outer plexiform layer; ELM, external limiting membrane; PR1, photoreceptor inner segments; PR2, photoreceptor outer segments; RPE, retinal pigment epithelium; BM, Bruch membrane. (**B**) Tomographic image obtained by DRI Triton SS-OCT and automatic segmentation was made by its IMAGEnet 6 Version software 1.221.14101 2014 Topcon Corporation. On the right margin are the layer abbreviations of the automated macular segmentation provided by DRI Triton SS-OCT software. The thickness of the RNFL, GCL+ and GCL++ protocols are described in the Materials and Methods section. Retinal nerve fiber layer thickness (RNFL) from the inner limiting membrane (ILM) line to the RNFL line. GCL+ thickness from the RNFL line to the inner plexiform layer (IPL) line and GCL++ thickness from the ILM line to the IPL line. IS/OS, inner/outer segments; RPE, retinal pigment epithelium; BM, Bruch membrane.

Each individual was imaged using a Spectralis SD-OCT^12^ (Heidelberg Engineering, Inc., Heidelberg, Germany) and *Deep* Range Imaging (DRI) Triton SS-OCT (Topcon Corporation, Tokyo, Japan). With the Spectralis SD-OCT, the volume fast macula scanning protocol was performed. The subject was asked to look into the internal fixation target, and Tru-Track eye tracking technology was used. Spectralis SD-OCT provides a circular macular map analysis, divided into nine sectorial thickness measurements in three concentric circles with diameters of 1, 3 (inner), and 6 (outer) mm forming the 9 areas corresponding to the ETDRS^14^. The central or subfoveal area (1 mm, R1), the 3 mm parafoveal ring with four areas, temporal inner (T1), superior inner (S1), nasal inner (N1), inferior inner (I1), and other four areas belonging to the 6 mm perifoveal ring: temporal outer (T2), superior outer (S2), nasal outer (N2), and inferior outer (I2) to study each macular layer thickness. The Spectralis software version was 6.8.1.0. Once the macular maps were segmented to obtain all retinal layers, the reference lines appeared automatically (Fig. 1); each retinal layer thickness was shown in the ETDRS area. The quality of the scans was checked, and poor-quality scans were rejected. Images should achieve at least 25 over 40 dB. With DRI Triton SS-OCT, a macular 6.0 × 6.0 mm three-dimensional scan was obtained, and automatic segmentation of each retinal layer was made by its IMAGEnet 6 Version software 1.22.1.14101 2014 Topcon Corporation (Fig. 1). DRI Triton SS-OCT includes the new SMARTTrack tool that enhances tracking, corrects for motion, and guides the operator to reduce potential errors while acquiring the images. Only eyes with good-quality scans defined as those with a signal strength ≥ 70/100 and without motion artefacts, involuntary saccades, or overt misalignment of decentration were included. DRI Triton SS-OCT provides the same circular macular map analysis as Spectralis SD-OCT, which is composed of the 9 areas corresponding to the ETDRS.

The Spectralis SD-OCT segments all the layers of the retina in the IRL and ORL. It also divides the IRL into 6 layers, the RNFL, the GCL, the IPL, the inner nuclear layer (INL), the outer plexiform layer (OPL) and the outer nuclear layer (ONL), which are the ones analysed in this study (Fig. 1).

The DRI Triton SS-OCT segments only part of the IRL, giving values of the RNFL, the GCL+ , which includes the GCL and the IPL, and the GCL++ , which includes the GCC (RNFL, GCL and the IPL) (Fig. 1).

To compare the DRI Triton SS-OCT measurements with the Spectralis SD-OCT measurements, we used DRI Triton SS-OCT protocols: the RNFL, GCL+ and GCL++ . We calculated the GCL+ thickness as the sum of the GCL and the IPL obtained by Spectralis SD-OCT, and the GCL++ was the sum of the values of the RNFL, the GCL and the IPL.

The exams with both OCT systems were performed on the same day and between 1:00 p.m. and 4:00 p.m. A single well-trained technician obtained all OCT images.

**Figure 2:**
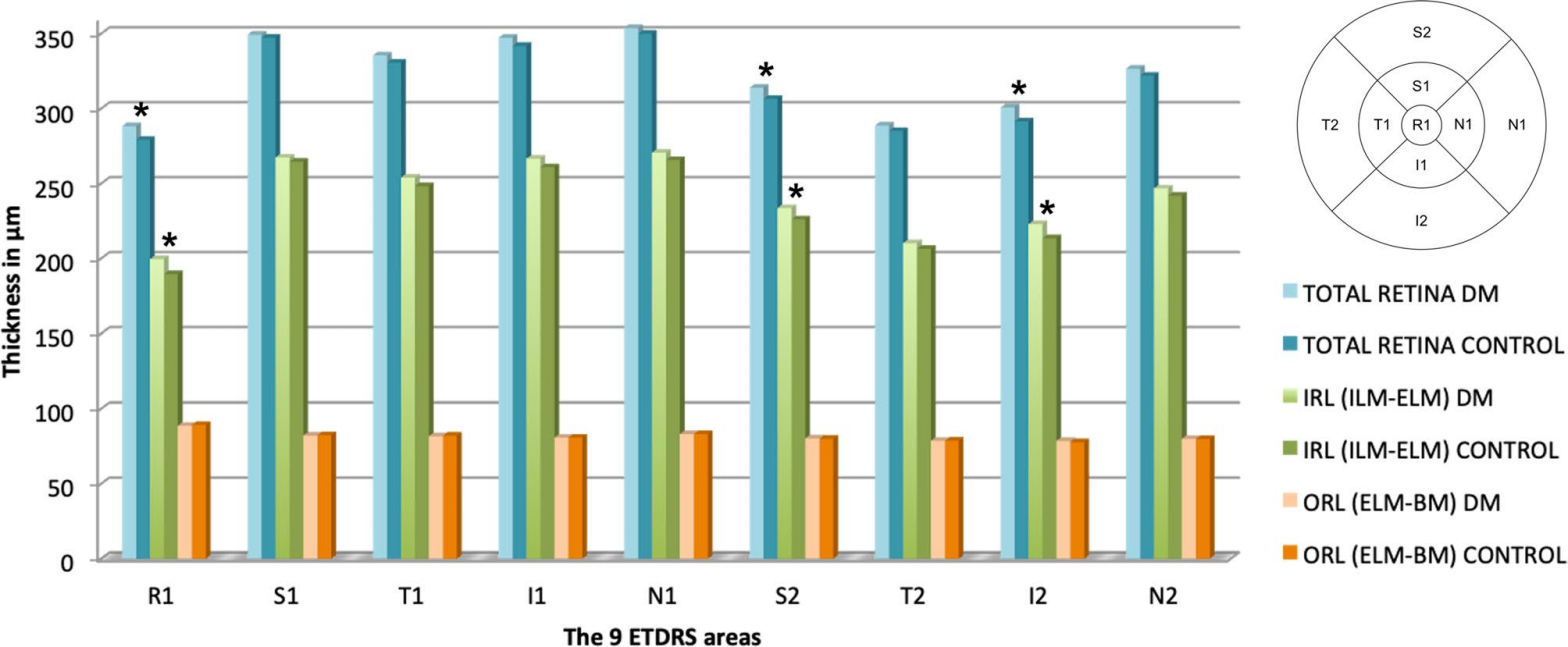
Spectralis SD-OCT measurements. Mean thickness per ETDRS area in μm and statistical significance with *p* < 0.05 marked with * of the total retina, the IRL (from the ILM to the ELM) and the ORL (from the ELM to the BM) in the diabetic and control groups. *DM* Diabetes mellitus, *ETDRS* Early Treatment of Diabetic Retinopathy Study, *ILM* Internal limiting membrane, *ELM* External limiting membrane, *BM* Bruch membrane, *IRL* Inner retinal layers, *ORL* Outer retinal layers, *R1* Central, *S* Superior, *T* Temporal, *I* Inferior, *N* Nasal, 1 corresponds to the values of the 3 mm parafoveal inner circle, and 2 corresponds to the values of the 6 mm peripheral outer circle.

**Figure 3:**
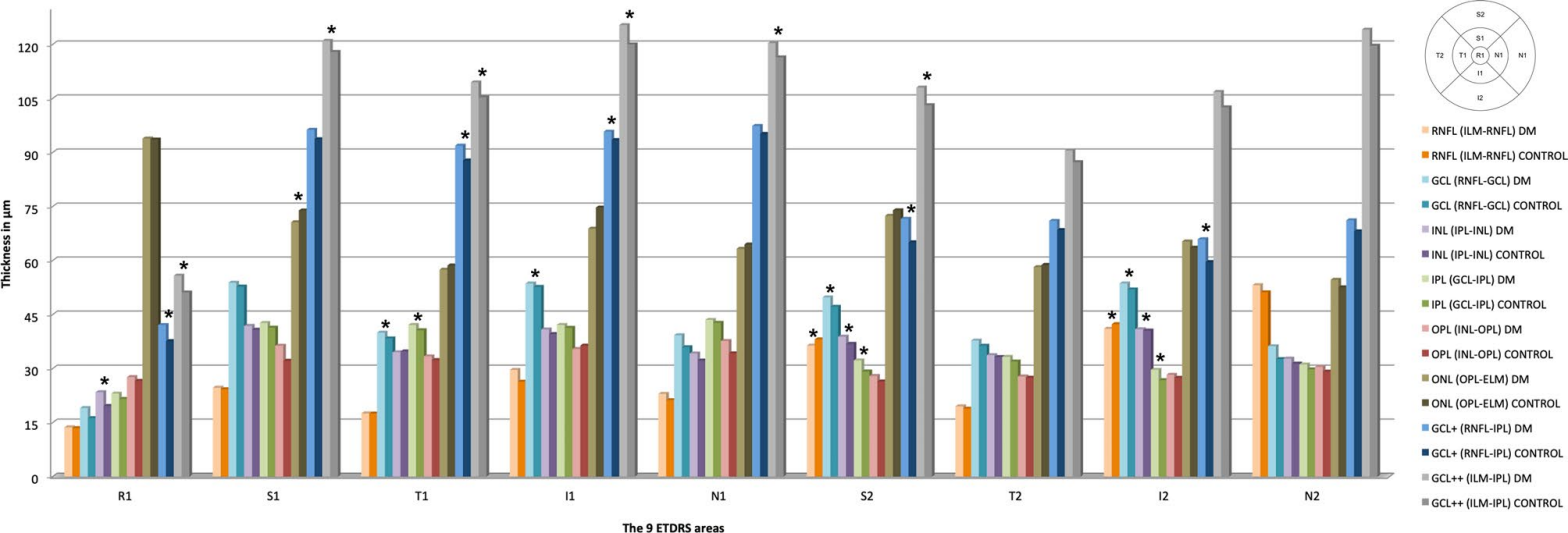
Spectralis SD-OCT mean thickness per ETDRS area is in μm, and statistical significance with *p* < 0.05 is marked with * of the RNFL (from the ILM to the RNFL), GCL (from the RNFL to the GCL), IPL (from the GCL to the IPL), INL (from the IPL to the INL), OPL (from the INL to the OPL), ONL (from the OPL to the ELM), GCL+ (from the RNFL to the IPL, adding GCL and IPL) and GCL++ (GCC) in the diabetic and control groups. *DM* Diabetes mellitus, *ETDRS* Early Treatment of Diabetic Retinopathy Study, *RNFL* Retinal nerve fiber layer, *ILM* Inner limiting membrane, *GCL* Ganglion cell layer, *IPL* Inner plexiform layer, *INL* Inner nuclear layer, *OPL* Outer plexiform layer, *ONL* Outer nuclear layer, *ELM* External limiting membrane, *R1* Central, *S* Superior, *T* Temporal, *I* Inferior, *N* Nasal, 1 corresponds to the values of the 3 mm parafoveal inner circle, and 2 corresponds to the values of the 6 mm peripheral outer circle.

**Figure 4:**
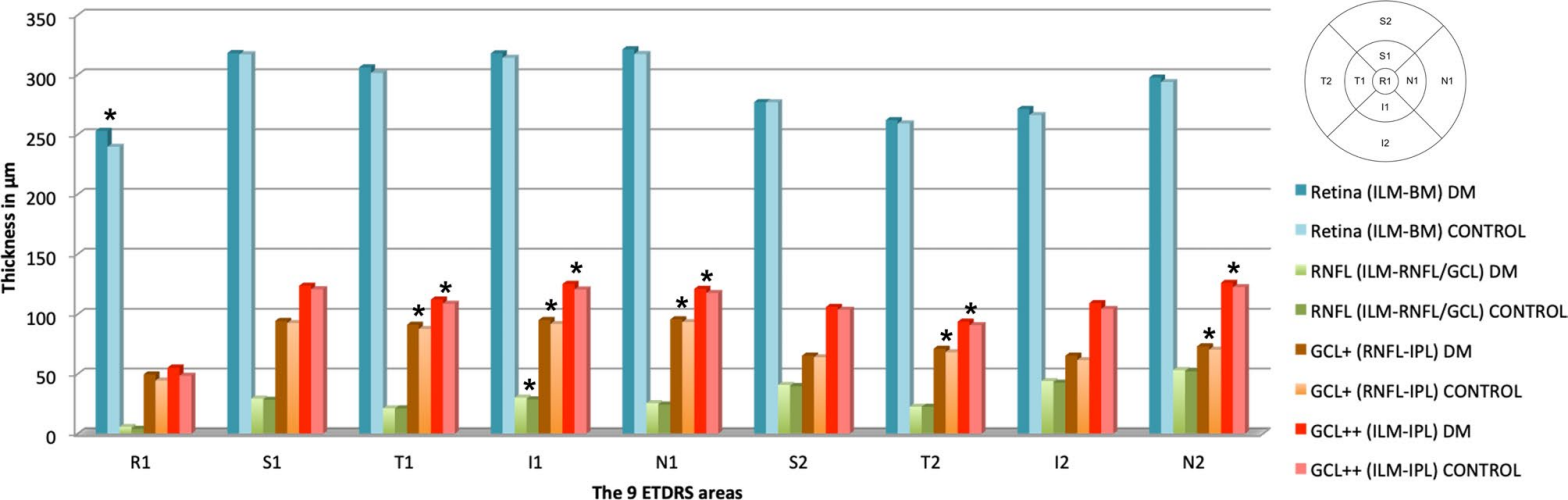
DRI Triton SS-OCT. Mean thickness per ETDRS area is in μm, and statistical significance with *p* < 0.05 is marked with * of the total retina (from the ILM to the BM), RNFL (from the ILM to the RNFL), GCL+ (from the RNFL to the IPL) and GCL++ (from the ILM to the IPL) in the diabetic group and in the control group. *DM* Diabetes mellitus, *ETDRS* Early Treatment of Diabetic Retinopathy Study, *RNFL* Retinal nerve fiber layer, *GCL* Ganglion cell layer, *BM* Bruch membrane, *ILM* Inner limiting membrane, *IPL* Inner plexiform layer, *R1* Central, *S* Superior, *T* Temporal, *I* Inferior, *N* Nasal, 1 corresponds to the values of the 3 mm parafoveal inner circle, and 2 corresponds to the values of the 6 mm peripheral outer circle.

**Figure 5:**
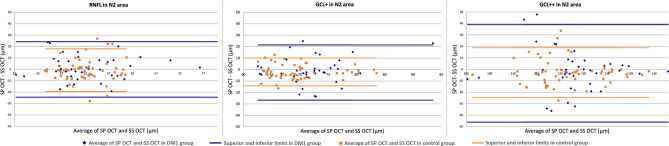
Blandt–Altman plots for the RNFL (from the ILM to the RNFL), the GCL+ (from the RNFL to the IPL) and the GCL++ (from the ILM to the IPL) average thickness between SD-OCT and SS-OCT in the nasal outer area N2. In each plot, corresponding layer is represented for the DM1 group in blue and for the control group in orange. *RNFL* Retinal nerve fiber layer, *GCL* Ganglion cell layer, *ILM* Inner limiting membrane, *IPL* Inner plexiform layer, *SD-OCT* Spectral domain optical coherence tomography, *SS-OCT* Swept source optical coherence tomography.

The measurements of the variables to be studied were recorded in an Excel database (Microsoft Office Excel 2011, Microsoft Corporation) and also the elaboration of Figs. 2, 3, 4 and 5. Statistical analysis was performed using the Statistical Package for the Social Sciences (SPSS 20, SPSS Inc., IBM Corporation, Somers, NY, USA). Normal distribution of the values was studied with the Kolmogorov–Smirnov test. As they did not have a normal distribution, the non-parametric Kolmogorov–Smirnov test was used to compare variables of the thickness of the different retinal layers between the two independent groups, such as the control group and the DM1 group. A *p* value < 0.05 was considered statistically significant.

### Ethics declarations

The experimental protocol was approved by the local Ethics Committee for Clinical Research of Aragon (CEICA 18/2017), and the evaluation was conducted in accordance with the principles of the Helsinki Declaration.

### Consent to participate

Detailed informed consent forms were obtained from each participant.

### Consent to publish

Consent for publication was given by each author and participant.

## Results

The mean age of the 90 DM1 patients was 41.52 ± 13.05 years (22–65) and 42.41 ± 13.56 years (26–68) for the 60 healthy controls, without age differences (*p* = 0.361). DM1 patients were well controlled, with a mean glycosylated haemoglobin (HbA1c) of 7.76 ± 1.06%. The mean DM1 evolution time was 24.88 ± 8.42 years (range 9–40 years).

Both groups had no differences in their AL (*p* = 0.908), anterior chamber depth (ACD) (*p* = 0.999) or refractive errors (*p* = 0.394).

### Functional results

BCVA taken with ETDRS tests for 100% contrast was very similar between the two groups (*p* = 0.91). When assessing the ETDRS with a 2.5% contrast, the BCVA in the DM1 group had lower values without reaching statistically significant differences (*p* = 0.24), but in the ETDRS with a contrast of 1.25%, statistically significant differences were found (*p* = 0.03), with the lowest BCVA in the DM1 group. In the color vision evaluation with the Farnsworth-Munsell 15D CVR test, worse results were observed in the DM1 group, but within the normal limits and with no differences in any of the indexes (Table 1).

**Table 1:** Mean and standard deviation (SD) of the visual function parameters obtained in the control group and in DM1 patients and their comparison (*p* value).

		Control group (mean ± SD)	DM patients (mean ± SD)	*p*
BCVA (logMAR)	ETDRS at 100%	− 0.113 ± 0.097	− 0.13 ± 0.11	0.91
	ETDRS at 2.5%	+ 0.21 ± 0.18	+ 0.35 ± 0.16	0.24
	ETDRS at 1.25%	+ 0.32 ± 2.27	+ 0.35 ± 2.87	0.03*
IOP (mmHg)		16.59 ± 2.27	16.82 ± 2.87	0.15
Farnsworth Munsell Color Test 15D	CCI	1.00 ± 0.00	1.04 ± 0.13	0.98
	AC CCI	1.00 ± 0.00	1.03 ± 0.19	0.98
	C-index	1.00 ± 0.00	1.05 ± 0.19	0.86
	S-index	1.48 ± 0.00	1.49 ± 0.09	0.98
	Confusion angle	61.5 ± 0.00	61.73 ± 7.68	0.98

The differences that reached statistical significance (*p* < 0.05) are indicated with *.

*DM1* Diabetes mellitus, *SD* Standard deviation, *BCVA* Best corrected visual acuity, *IOP* Intraocular pressure, *ETDRS* Early Treatment of Diabetic Retinopathy Study, *CCI* Color confusion index, *AC-CCI* Age-corrected color confusion index, *C-index* Confusion index, *S-index* Selectivity index.

### SD-OCT results

Comparing total retinal thickness between the DM1 group and the control group measured by Spectralis SD-OCT and its macular protocol, we only found differences in the central area (R1, *p* = 0.030) and the vertical perifoveal areas (S2: *p* = 0.004 and I2: *p* = 0.009, respectively), with greater thicknesses in the DM1 group. The R1 thickness values were 288.28 ± 28.59 μm vs 279.28 ± 16.36 μm in the DM1 group vs control group, respectively. In the S2 area, the values were 313.91 ± 20.65 μm and 306.57 ± 16.07 μm in the DM1 group and control group, respectively, and in the I2 area, the values were 300.76 ± 22.68 μm vs 291.37 ± 14.81 μm in the DM1 group and control group, respectively. Dividing the retina into internal (IRL) and external (ORL) layers, we observed that in the three described areas (R1, S2 and I2), these differences belonged to the IRL, with no changes in the ORL thicknesses (Fig. 2).

We studied each IRL layer to evaluate the observed differences, including the RNFL, GCL, IPL, INL, OPL and ONL in the different ETDRS areas (Fig. 3). The majority of these retinal layer thickness values were higher in the DM1 group than in the controls, but the differences were observed in the IRL in the central area (R1) belonging mostly to the INL (*p* = 0.003), where there are only cones or Müller cell nuclei.

In the vertical peripheral areas (S2 and I2), we observed that the IRL differences reached statistical significance in the four innermost layers. We found thickness differences in the RNFL (S2, *p* = 0.012; I2, *p* = 0.044), GCL (S2, *p* < 0.001; I2, *p* = 0.002), IPL (S2, *p* = 0.004; I2, *p* = 0.023) and INL (S2, *p* = 0.003; I2, *p* < 0.001).

The GCL was the layer with higher differences between groups observed in the inner parafoveal ring, where their cell bodies are located. The T1 and I1 areas had higher thicknesses in the DM1 group (T1: 39.98 ± 4.58 μm vs 38.38 ± 4.00 μm with *p* < 0.001 and I1: 53.62 ± 5.99 μm vs 52.67 ± 4.41 μm with *p* = 0.030, in DM1 patients vs controls, respectively). However, there were no differences in the ONL between the groups. Differences were also found in the S1 area of the OPL (*p* < 0.001) and in the T1 area of the IPL (*p* = 0.014), with higher values in the DM1 patients (Fig. 3).

Calculating GCL+ and GCL++ with Spectralis SD-OCT, differences were found in the GCL+ in the R1 (*p* = 0.011), T1 (*p* < 0.001), I1 (*p* = 0.024), S2 (*p* < 0.001) and I2 (*p* < 0.001) areas. For GCL++ , the only areas with no differences were the peripheral T2 (*p* = 0.273), I2 (*p* = 0.190) and N2 (*p* = 0.162) areas (Fig. 3).

### SS-OCT results

Total retinal thickness using the DRI Triton SS-OCT had significant differences at the central area (R1), achieving values of 253.11 ± 28.36 μm vs 239.77 ± 22.91 μm in DM1 vs control (*p* = 0.014), respectively, which were the highest values in the DM1 group as already described with Spectralis SD-OCT. The values obtained with Spectralis SD-OCT were 30–40 μm higher than those obtained with DRI Triton OCT. However, unlike the results obtained with the Spectralis SD-OCT, for DRI Triton SS-OCT, there were no statistically significant differences in either S2 or I2 areas.

When studying the RFNL with DRI Triton SS-OCT, differences were observed in the I1 area with values of 30.20 ± 3.70 μm vs 28.78 ± 2.04 μm in the DM1 group vs the control group, respectively (*p* = 0.016), where it is barely thick and probably due to the high standard deviation present in the DM1 group (Fig. 4).

Using the GCL+ and GCL++ protocols, statistically significant differences were observed in all 3 mm ring areas except S1.

When studying the 6 mm perifoveal ring with both protocols, we found significant differences in the horizontal areas of the GCL+ (T2, 71.13 ± 7.85 μm vs 68.21 ± 6.52 μm, *p* = 0.017 and N2: 73.08 ± 9.35 μm vs 70.25 ± 6.49 μm, *p* = 0.004 in the DM1 and control groups, respectively) and in the GCL++ (T2, 93.75 ± 9.12 μm vs 90.77 ± 7.50 μm, *p* = 0.017 and N2: 126.29 ± 14.54 μm vs 122.55 ± 9.83 μm, *p* = 0.004, in the DM1 and control groups, respectively).

### SD-OCT versus SS-OCT results

To compare the DRI Triton SS-OCT measurements with the Spectralis SD-OCT measurements, we used DRI Triton SS-OCT protocols: the RNFL, GCL+ (sum of GCL and IPL with Spectralis SD-OCT) and GCL++ (sum of RNFL, GCL and IPL with Spectralis SD-OCT). We did not find the same differences with both devices, especially in the values of the RNFL and in the outer ring values (Figs. 3, 4).

The RNFL thickness values were the ones with the highest differences between the two devices (*p* < 0.001). The studied layers had very low thickness values, making segmentation difficult. These differences disappeared closer to the optic nerve in the nasal perimacular areas in both groups (N2: *p* = 0.792 in controls and *p* = 0.512 in DM1) since the RNFL reached its greatest thickness.

The GCL+ and GCL++ values in the control group were quite similar between the two OCT systems, with differences only in the R1 (*p* < 0.001) and I2 areas (*p* = 0.024) of the GCL+ and in both temporal quadrants (T1: *p* = 0.004 and T2: *p* = 0.014) for the GCL++ ; in both protocols, the thicknesses were higher with DRI Triton SS-OCT. In the DM1 group, we found differences in GCL+ and GCL++ between devices in a greater number of areas. For the GCL+ , we obtained differences in R1 (*p* < 0.001) and in all peripheral areas (S2: *p* < 0.001, I2; *p* = 0.037 and N2: *p* = 0.012), except in the temporal area (T2: *p* = 0.825). In contrast, in the GCL++ containing the RNFL, differences were observed in all the peripheral quadrants (S2: *p* < 0.001, T2: *p* = 0.001, I2: *p* < 0.001 and N2: *p* < 0.001) and in two of the parafoveal quadrants (T1: *p* < 0.001 and I1: *p* < 0.001) with thicker thicknesses in this layer with the DRI Triton SS-OCT. All the differences that reached statistical significance for the three protocols in the control group were repeated in the DM1 group.

Figure 5 shows the measurements of each layer and in the same area taken by both devices The comparison found predominantly agreements between the two OCTs.

## Discussion

In our study, we only found differences in visual function in the BCVA measurement with a contrast of 1.25% (*p* = 0.030). The BCVA with 100% contrast in our DM1 group without DR was good (− 0.13 ± 0.11 logMAR, *p* = 0.910), better than the BCVA found by Zhu et al. (0.10 ± 0.19 logMAR) in DM2; they also found no differences with the control group^15^. In another follow-up study, the initial BCVA in DM1 patients was − 0.03 ± 0.16 logMAR but worsened to 0.03 ± 0.20 logMAR 10 years later^16^. Good BCVA values were related to the mean age of the DM1 group (41.52 ± 13.05 years) and with acceptable glycaemic control (HbA1c, 7.76 ± 1.06%)^17^.

In patients with DR in DM1 and DM2, there is a decrease in CS, which may be independent of the involvement of VA^2,15^, but there is no agreement on whether this involvement begins before DR; if so, this fact would support an early degeneration of retinal neurons. Several studies found a decrease in CS in DM1 and DM2 without DR for all spatial frequencies, with higher differences while increasing the spatial frequency^2,3,18^. It has been suggested that the selective loss of the CS for higher frequencies could be a sign of parvocellular pathway dysfunction^1^, which comprises 80% of the ganglion cells that are responsible for contrast transmission and color^19^.

Regarding color vision, in our study, we did not find differences between groups in any of the Farnsworth-Munsell 15D test indexes. Numerous studies have detected the presence of defects in the color vision of DM1 and DM2 patients with DR in the blue-yellow axis (tritanopia)^20,21^. Color vision defects have also been described in DM1 and DM2 without DR^1,22^, suggesting that it may be an early manifestation of the neuronal dysfunction of DM, with cone effects in the absence of visible microvascular changes^23,24^.

OCT has become an important tool to study the retina in DM patients in early stages and to control the changes that occur therein throughout the disease^25^. In some of the studies in diabetic patients using OCT, there seemed to be retinal thickening as an early DR sign, without the existence of diabetic macular oedema^25,26^. Retinal thickness variations could be related to modifications in the blood-retinal barrier^27–29^, changes in vascular pericytes and endothelial cells^27,30^, or in glial cells, preferably Müller cells. Other studies have demonstrated neuroglial modification caused by sustained hyperglycaemia^27,31,32^, with different suggestions; some authors discuss that there may be neuroglial loss with a decrease in layer thickness^32^, or on the contrary, Müller cell hyperreactivity with an increase in central area thickness^33^.

In our study, the average total retinal thickness in the 9 ETDRS areas was thicker in the DM1 patients than that in the control group. Lattanzio et al. reported similar results, with a thickness increase measured with OCT in both DM1 and DM2 patients, being thicker with the DR degree, but they did not review the time of DM evolution or metabolic control^34^. The thickness increase once the DR is established is a proven fact given the diffusion from the vessels due to the rupture of the internal blood-retinal barrier, causing macular damage^27–29^.

We found thickness differences in the total retina with Spectralis SD-OCT between the control and the DM1 group in the central area (R1, *p* = 0.030) and in vertical perifoveal areas (S2, *p* = 0.004 and I2, *p* = 0.009). With the DRI Triton SS-OCT, we also found differences in the central area (R1, *p* = 0.014), with DM1 values higher than those of the control group. Sanchez-Tocino et al.^26^ studied DM patients without DR with similar characteristics and found an increase in the central area (R1) of the DM group. These authors also comment that there were no differences in any of the macular parameters of their study between patients without DR and patients with DR without clinically significant diabetic macular oedema. Dhasmana et al. found a higher foveal thickness in DM2 patients with DR compared to those without DR and thicker foveal thickness than the control group without reaching differences between patients without DR and healthy subjects^35^.

The fovea (R1) recorded the lowest thickness with both devices, in addition to the greater thickness difference between groups in the total retina (differences between DM1 and controls of 9 μm and 13.34 μm in Spectralis SD-OCT and DRI Triton SS-OCT, respectively, superior for the DM1 group) followed by the peripheral temporal (T2) ones.

Retinal thickness was higher with Spectralis SD-OCT than with DRI Triton SS-OCT, which is related to their differences in their established limits. With Spectralis SD-OCT, retinal delimitation reaches the BM, while DRI Triton SS-OCT delimits the retina to the upper limit of the RPE^36,37^. Other factors are their differences in technologies, wavelengths and software^38,39^. It would be convenient to incorporate a conversion formula for comparing thickness measurements with different OCTs^40^.

We found that the total retinal areas measured by Spectralis SD-OCT that showed differences were justified by changes in the IRL (R1, S2 and I2), while no modifications were found in the ORL thicknesses (Fig. 2). Ciresi et al.^41^ also found an increase in thickness in DM1 without DR in both the central (R1) and 6 mm peripheral areas. We segmented and compared each layer separately, and we only found differences in the INL central area (R1) (*p* = 0.003). There are different reasons that justify these findings, such as some inclination in the tomographic image, segmentation error due to the low thickness values at this level and the difficulty of manual modifications or changes at Henle's fiber layer because at this level, we only have photoreceptors and Müller cell nuclei.

Looking at the 3 mm parafoveal ring and analysing the different IRL layers with Spectralis SD-OCT, the differences in T1 and I1 belong to the GCL. Regarding the GCL-IPL and GCC, we found differences in the 3 mm parafoveal ring in the T1, I1 and N1 areas with both protocols and with both devices (excluding N1 for DRI Triton OCT). The ganglion cell bodies are located in this 3 mm ring, and if there are modifications in the GCL, thickness changes will occur here.

For the 6 mm perifoveal ring with Spectralis SD-OCT, the differences between the DM1 and the control group were observed in the vertical areas (S2 and I2) in the four innermost layers: RNFL, GCL, IPL and INL. We again objectify the low thickness values of all these layers; the RNFL is mainly located in the 6 mm peripheral circle where it can undergo major thickness changes.

There were significant differences between groups with DRI Triton SS-OCT, in the 6 mm peripheral ring, and in the horizontal areas for both GCL+ and GCL++ protocols.

Analysing the results obtained by both devices in each group, we found important thickness differences, especially in the RNFL thickness values, with statistical significance in 8 of the 9 ETDRS areas. These differences disappeared in the peripheral nasal area (N2), where the RNFL reaches its greatest thickness close to the optic nerve in both groups. Therefore, the RNFL values are not comparable for both OCT systems and are more reliable when thickness values increase. Both GCL-IPL and GCC were quite similar between the two OCT systems. Only significant differences were obtained for the GCL+ in R1 and I2 and for the GCL++ in both temporal areas (T1 and T2). This fact suggests that although layers are analysed in an individual way, there are differences between them when values increase, and these differences tend to disappear, as already described with the RNFL thickness. We found more differences in the DM group in the GCC, including the RNFL, with both protocols (GCL+ and GCL+ +), and for both groups, larger thicknesses were found with DRI Triton SS-OCT than Spectralis SD-OCT. With these data, we can speculate that the GCL+ and the GCL++ could be compared between OCT devices in healthy subjects, since the differences are minimal, but in pathological patients caution is necessary since the alterations in the different retinal layers can be segmented differently with each OCT giving differences in more ETDRS areas when comparing the values.

The superior RNFL loss in diabetic patients has also been attributed to less perfusion in the superior retina and optic nerve head, which could generate greater ischaemia that structurally damages ganglion cells^42^.

Histological studies have identified highly suggestive retinal neurodegeneration changes (loss in RNFL and GCL and increase in glial cells) in animal models or in postmortem studies of diabetic donors^13,43^.

This study demonstrates that in DM1, the GCL is vulnerable to progressive damage before the appearance of microvascular DR. We found some studies in DM1 and DM2^44–47^ in which subjects with moderate or severe DR had a thinner GCL than subjects without DR. Vujosevic et al.^48^ found this same decrease in GCL in DM1 and DM2 subjects who had no obvious DR lesions, which was reflected in thinning at the RNFL level. Vujosevic et al. using DRI-triton OCT, and after adjusting for age and DM duration^49^, found that GCL+ (49.4 μm vs. 43.6 μm, *p* = 0.0099) and GCL++ complex (57.2 μm vs. 50.5 μm, *p* = 0.0367) thicknesses in R1 were significantly higher in patients with DM1 than in patients with DM2 without DR, but in both DM groups were thinner than controls; GCL++ complex in the inner ring was thinner in patients with DM versus controls. Srinivasan et al.^50^, using SD-OCT, found that DM2 patients had significantly reduced full retinal thickness in the parafovea and perifovea and the reduction of the RNFL and GCC thickness was higher than DM1 patients. Scarinci et al.^51^, recently reported a thinning of the GCL layer in patients with DM1 and no DR with SD-OCT. The exact mechanisms for IRL thickness loss, a fact revealed in our study after 8 years of evolution in the same sample of DM1 patients^12^ and compatible with the neurodegeneration theory, are not clear but have been related to less perfusion and higher metabolic demands of the internal retina that make it more vulnerable to diabetes-induced metabolic stress^44^. Other factors could modify the retinal structure, RNFL thinning has been attributed to the microvascular changes such as leukostasis, vascular obliterations, changes basal membrane^52–54^. Homocysteine levels have a positive correlation with the thinning of the RNFL^55^. Thinning of the RNFL has also been correlated with the severity of the disorganization of the inner retinal layers (DRIL) in diabetic patients^56^ and an increase of serum levels of VEGF or ICAM1 have been related to the degree of the DR or the disruptions in the ELM and ellipsoid zone^57^.

We did not find ONL differences between groups measured with Spectralis SD-OCT.

In conclusion, long-term DM1 patients with no DR signs maintained visual function, except for a loss in 1.25% ETDRS contrast. Both Spectralis SD-OCT and DRI Triton SS-OCT can detect changes in the macular thickness of the different retinal layers.

Patients with DM1 without DR have thickness changes in the IRL measured in the ETDRS areas, especially in the ganglion cell bodies located in the GCL at the parafoveal level, generating damage of the RNFL in the peripheral areas. This RNFL thinning and the retinal neurodegeneration could be a possible strategy for treating DM patients slowing down the development of the DR.

There were no differences between controls and diabetic patients in ORL at the macular level. New studies on changes at the structural OCT level and other changes such as modifications at the OCTA level are necessary to confirm and justify this finding.

## Data Availability

All the necessary data are included in the manuscript.
